# Supplementary material for: Growth-altering microbial interactions are responsive to chemical context
Source: PLoS One. 2017 Mar 20;12(3):e0164919. doi: 10.1371/journal.pone.0164919 (PMC5358735; doi:10.1371/journal.pone.0164919)
Supplement: S1 Supplemental Material — (DOCX) [file pone.0164919.s002.docx]

**Full Title:** Growth-Altering Microbial Interactions Are Responsive to Chemical Context

**Short Title:** Microbial Interactions Responsive to Context

Angela Liu^1¶^, Anne M. Archer^2¶^, Matthew B. Biggs^1*^, Jason A. Papin^1*^

1. Department of Biomedical Engineering, University of Virginia, Charlottesville, Virginia
2. Department of Biology, University of Virginia, Charlottesville, Virginia

**¶** These authors contributed equally to this work.

* Co-corresponding authors

Email: mb3ad@virginia.edu (MB), papin@virginia.edu (JP)


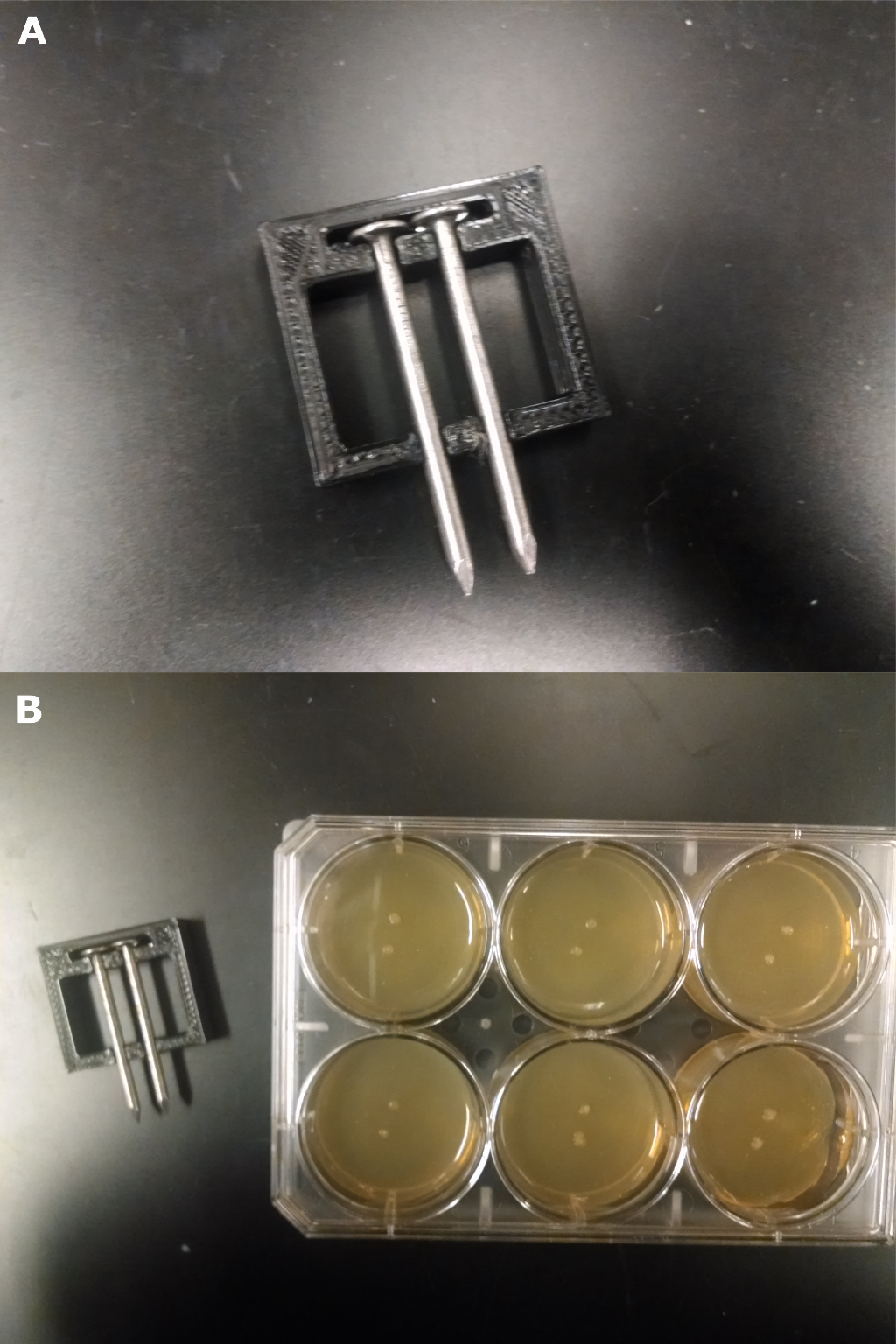


**Supplemental Figure A. The colony stamping mechanism**. A) A picture of the 3D-printed stamping mechanism loaded with two 1 ½ inch box nails (size 4D). B) The stamping mechanism beside a standard 6-well plate containing BHI agar and bacterial colonies.

**Supplemental Figure B.**
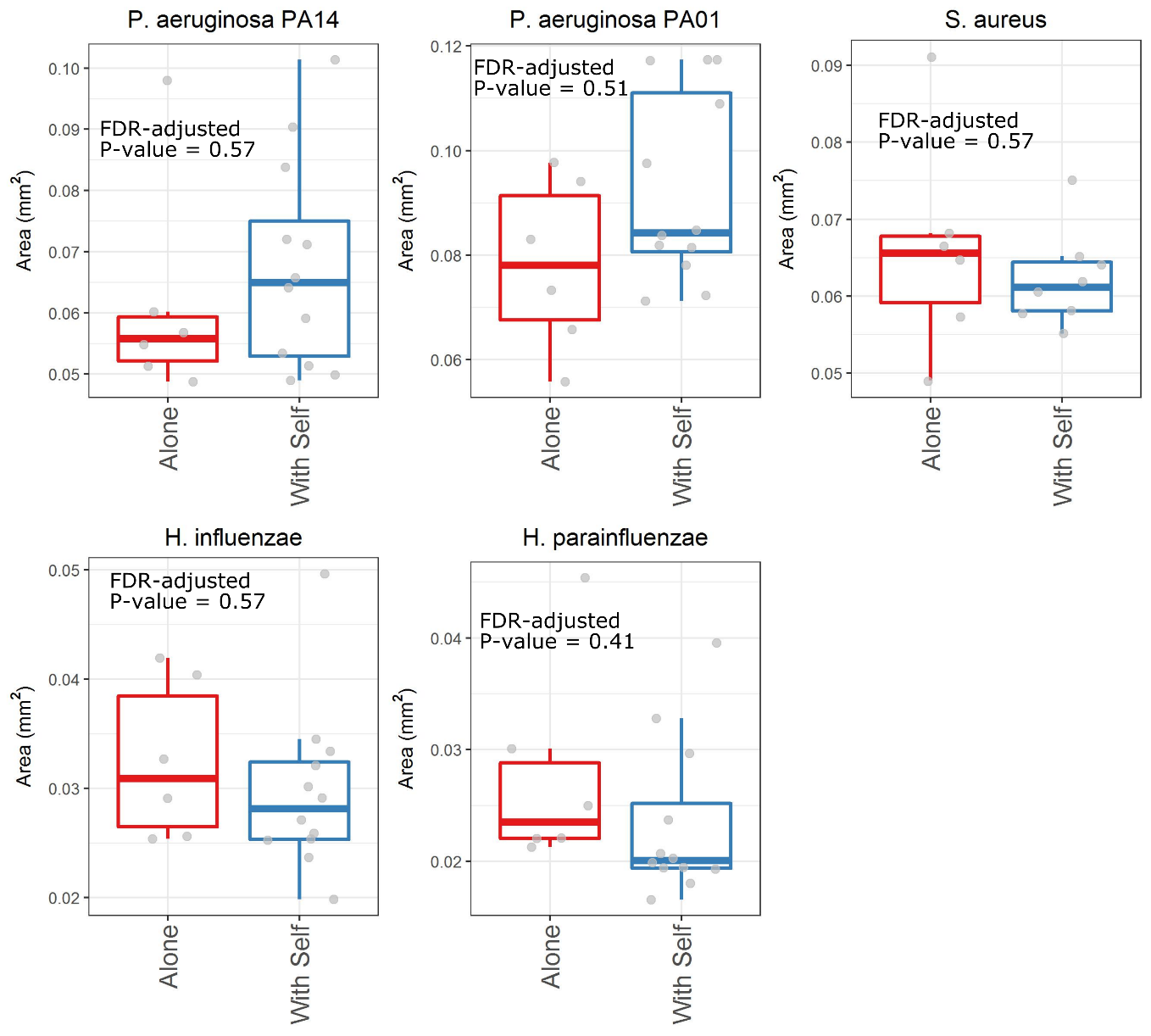
**Each species grown alone and next to itself.** Six replicates were grown of each condition (resulting in 12 replicates of the “with self” condition). By two-sided Wilcoxon signed-rank test, no p-values were significant initially (p < 0.05), and after FDR correction all p-values were greater than 0.4. Note that some outliers are plotted outside the bounds of these plots.

**Supplemental Figure C.**
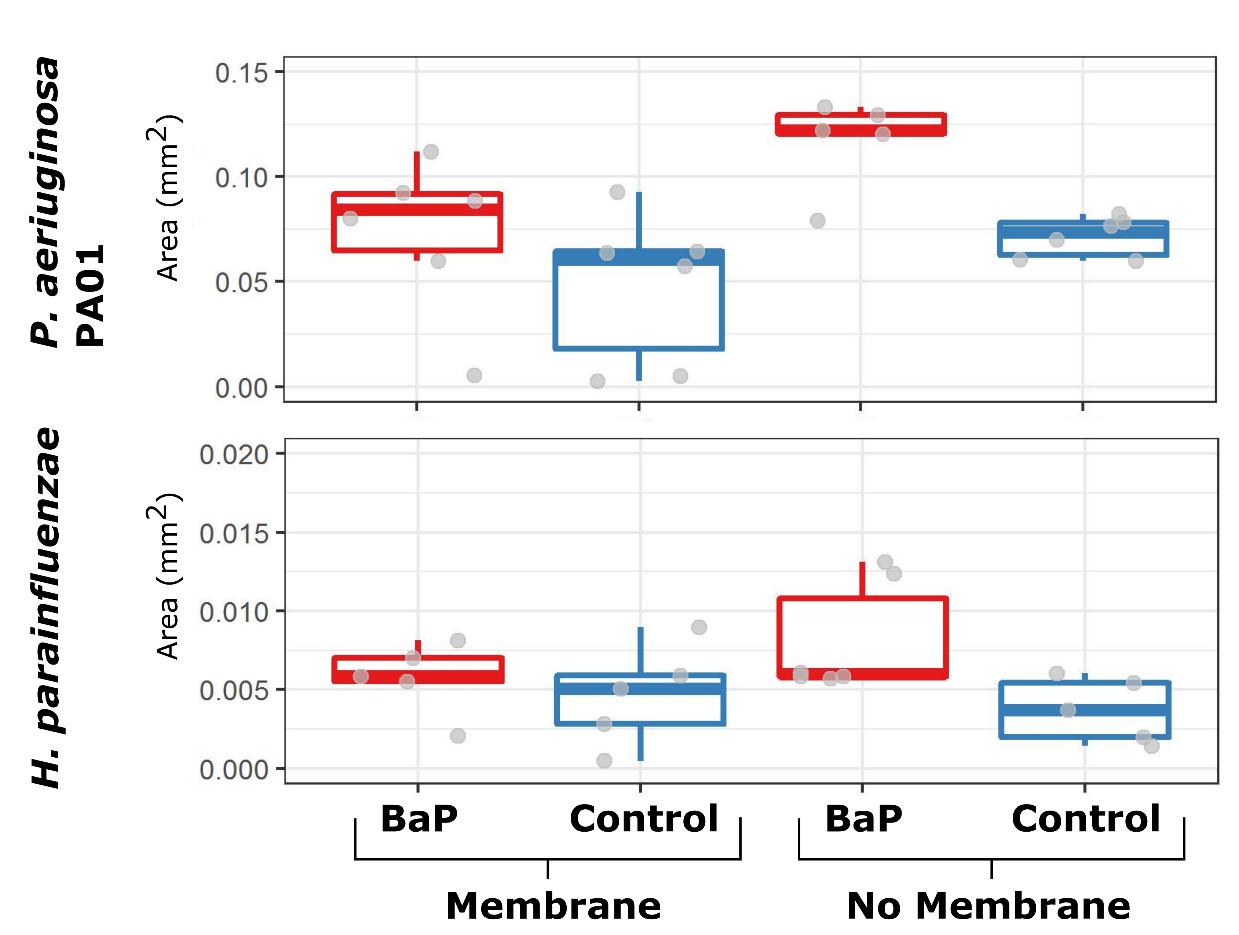
**The influence of a semi-permeable membrane on species interactions.** *P. aeruginosa* PA01 was inoculated next to *H. parainfluenzae* on BHI agar with and without BaP. The conditions were replicated with the addition of a 0.1 µm VCTP membrane (EMD Millipore) separating the two species. There were six replicates in each experimental condition. In this case, there was no statistically significant difference between conditions with and without the membrane (two-sided Wilcoxon signed-rank test, p< 0.05). However, the addition of a membrane is a potential tool for discriminating between contact-dependent and –independent interactions.
